# Multi-modal survey of Adélie penguin mega-colonies reveals the Danger Islands as a seabird hotspot

**DOI:** 10.1038/s41598-018-22313-w

**Published:** 2018-03-02

**Authors:** Alex Borowicz, Philip McDowall, Casey Youngflesh, Thomas Sayre-McCord, Gemma Clucas, Rachael Herman, Steven Forrest, Melissa Rider, Mathew Schwaller, Tom Hart, Stéphanie Jenouvrier, Michael J. Polito, Hanumant Singh, Heather J. Lynch

**Affiliations:** 1 Department of Ecology and Evolution, 113 Life Sciences, Stony Brook University, Stony Brook, NY 11794 United States; 2 Department of Applied Ocean Physics and Engineering, Woods Hole Oceanographic Institution, Woods Hole, MA 02543 United States; 3 Department of Mechanical Engineering, Massachusetts Institute of Technology, Cambridge, MA 02139 United States; 4 Department of Zoology, South Parks Road, Oxford, OX1 3PS United Kingdom; 5 Natural Resources and the Environment, James Hall, University of New Hampshire, Durham, NH 03824 United States; 6 Antarctic Resource, Inc., 303 S. Broadway, Suite 200-190, Denver, CO 80209 United States; 7 Department of Oceanography and Coastal Sciences, Louisiana State University, Baton Rouge, 70803 United States; 8 Biology Department, Woods Hole Oceanographic Institution, Woods Hole, MA United States; 9 Centre d’Etudes Biologiques de Chizé, UMR 7372 Centre National de la Recherche Scientifique/Univ La Rochelle, Villiers en Bois, France

## Abstract

Despite concerted international effort to track and interpret shifts in the abundance and distribution of Adélie penguins, large populations continue to be identified. Here we report on a major hotspot of Adélie penguin abundance identified in the Danger Islands off the northern tip of the Antarctic Peninsula (AP). We present the first complete census of *Pygoscelis* spp. penguins in the Danger Islands, estimated from a multi-modal survey consisting of direct ground counts and computer-automated counts of unmanned aerial vehicle (UAV) imagery. Our survey reveals that the Danger Islands host 751,527 pairs of Adélie penguins, more than the rest of AP region combined, and include the third and fourth largest Adélie penguin colonies in the world. Our results validate the use of Landsat medium-resolution satellite imagery for the detection of new or unknown penguin colonies and highlight the utility of combining satellite imagery with ground and UAV surveys. The Danger Islands appear to have avoided recent declines documented on the Western AP and, because they are large and likely to remain an important hotspot for avian abundance under projected climate change, deserve special consideration in the negotiation and design of Marine Protected Areas in the region.

## Introduction

Monitoring populations is essential to species conservation, and can be used to identify threats or changes in conservation status. Indeed, central to the Convention on Biological Diversity and related conservation measures is the assumption that we can quantify species diversity, abundance, and geographic distribution^1,2^. Numerous studies now suggest that Adélie penguin (*Pygoscelis adeliae*) populations are undergoing dramatic shifts in abundance, with marked declines along most of the Western Antarctic Peninsula (WAP) and associated sub-Antarctic Islands^3–8^ and sharp increases in the Ross Sea and Eastern Antarctica^8–14^. While the causal drivers of these changes remain unknown and may in fact vary across the continent, several studies have linked Adélie penguin population trends to changes in sea ice extent and concentration as well as changes in air temperature and precipitation patterns and their possible effects on prey availability^14–17^. Understanding the population dynamics of sentinel species, such as the Adélie penguin, will help illuminate the effects of climate change on less easily-studied components of the ecosystem^18,19^. Much of the concern regarding climate-driven changes has been focused on the WAP and South Shetland/South Orkney Islands, where Adélie penguin populations have declined sharply (~70%) over the last several decades^20,21^. Along the WAP, the northern end of Marguerite Bay (67°30′S) represents a clear boundary that divides areas of Adélie penguin population decline in the north from areas where abundances are either stable or increasing to the south^22^. Far less is known about Adélie penguin populations along the northern and eastern portions of the AP, a region perhaps more closely tied to the Weddell Sea in terms of climate and sea ice production than to the dynamics of the WAP^23,24^.

**Figure 1:**
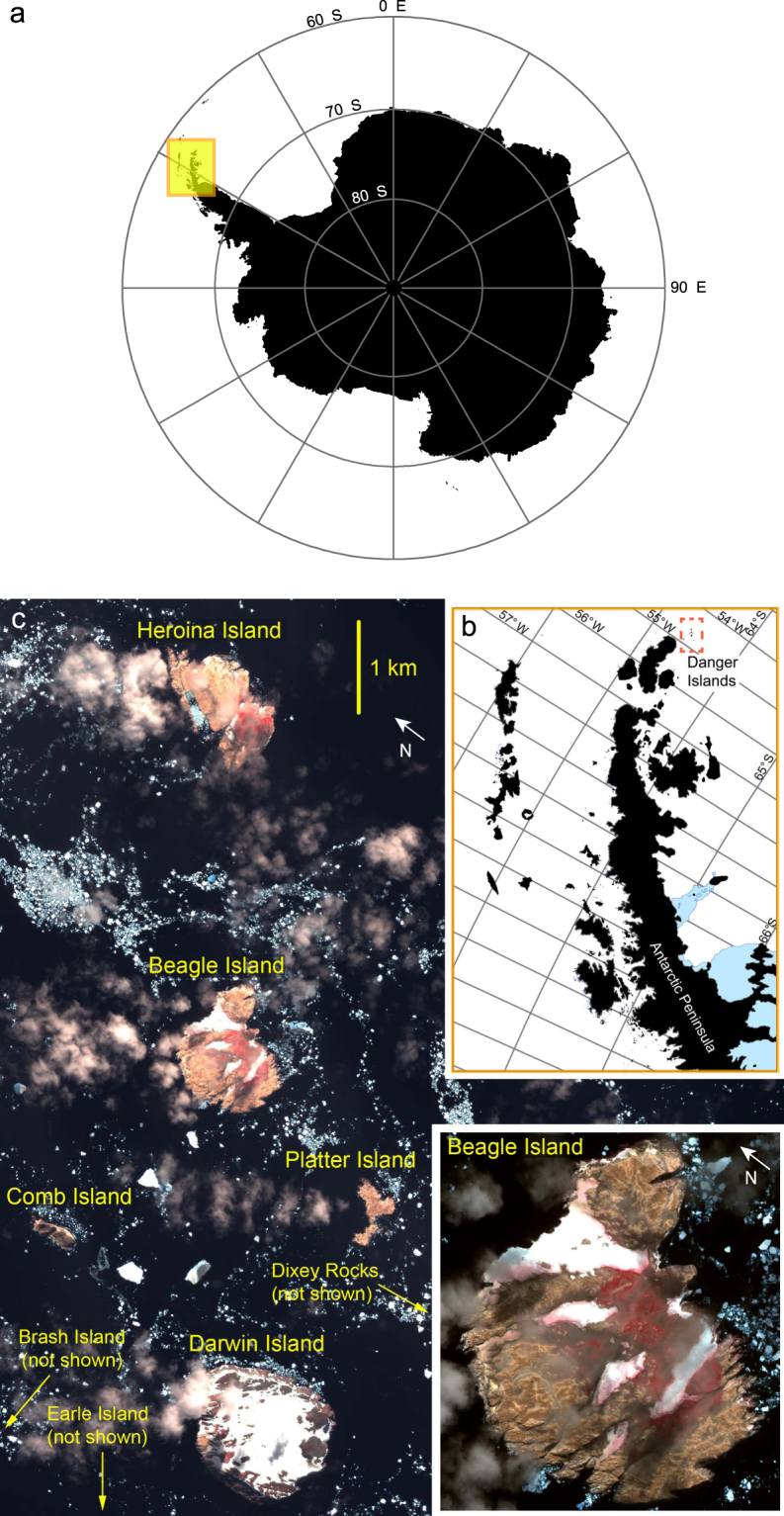
(**a**) Map showing the location of the Antarctic Peninsula and (**b)**, the location of the Danger Islands group on the Antarctic Peninsula, both created using ESRI ArcMap 10.0 (http://desktop.arcgis.com/en/arcmap/). (**c)** Quickbird image of the Danger Islands taken 22 January 2011 (©2018, DigitalGlobe).

The Danger Island archipelago is comprised of 9 islands stretching over approximately 35 km at the northernmost tip of the AP in the north-western Weddell Sea (Fig. 1). Despite their relative proximity to the WAP, which sees much of the tourist and fishing ship traffic in the Antarctic^25^, pack ice is common around the Danger Islands even in austral summer^26^. In fact, due to the currents of the Weddell Sea, which drive sea ice northward, access to the islands is precluded in most years. Heroína Island, at the northeast end of the archipelago, is the most frequently visited of the Danger Islands and yet hosts a median visitation rate of only one ship landing per year^27^. It is also the only island to date with a population estimate (285,000–305,000) derived from a ground survey of the island^28,29^. While a previous geological expedition^30^ noted the presence of Adélie penguins on all of the Dangers Islands (with the exception of Darwin Island, which was not visited) and several others were photographed (by M.R. and S.F.) from a passing vessel in 2008/09, the presence of Adélie penguins on several of these islands went largely unrecognized until a recent Landsat satellite survey of the Antarctic identified several large penguin colonies supporting what appeared to be nearly 200,000 Adélie penguin nests^31^. The (re)discovery of these populations, combined with evidence that the Danger Islands as a group supported a regionally-significant population, motivated an expedition to the area. In this paper we report on the first comprehensive seabird survey of the Danger Islands and describe a heretofore unrecognized Adélie penguin hotspot. We document a novel multi-modal survey comprised of ground surveys and imagery from both satellites and unmanned aerial vehicle (UAV) photographic surveys, the results of which were combined with historic aerial photographs to assess long-term change in the region. We also discuss the implications of this seabird hotpot for the design of Marine Protected Areas in the Antarctic Peninsula region.

## Results

Our survey found 751,527 (95^th^ CI = [710,103–792,443]) nesting pairs of Adélie penguins in the Danger Islands (Table 1). When combined with known information on abundance elsewhere in the region^21^, we estimate the Danger Islands contain 55% of all Adélie penguins in subarea 48.1 as defined by the Commission for the Conservation of Antarctic Marine Living Resources (CCAMLR). This subarea includes all of the western AP, the waters north of the AP to 60°S, and a portion of the north-western Weddell Sea west of 50°W. Without the Danger Islands, 48.1 contains 606,526 (95^th^ CI = [322,477–990,402]) Adélie penguin pairs^21^.

**Table 1:** Census summary. ‘N1’: $\le$5% error; ‘N2’$\pm$:10% error; ‘N4’: $\pm$50% error.

	Beagle^†^	Brash^†^	Comb^†^	Darwin^†^	Dixey Rock^†^	Earle^†^	Heroína	Platter^†^	Scud Rock^†^
*Pygoscelis adeliae*	284535^‡^ (N2)	94951^‡^ (N2)	12000^#^ (N4)	5804^#^ (N1)	0 (N1)	21071^‡^ (N2)	292363^‡^ (N2)	40803 (N1)	0 (N1)
*Pygoscelis papua*	0 (N1)	2270 (N1)	186 (N1)	0 (N1)	0 (N1)	847 (N1)	999 (N1)	223 (N1)	0 (N1)
*Pygoscelis antarctica*	0 (N1)	0 (N1)	0 (N1)	0 (N1)	0 (N1)	0 (N1)	27 (N1)	0 (N1)	0 (N1)
*Phalacrocorax atriceps*					0 (N1)	156 (N1)			0 (N1)

^†^First direct census of this location; ^‡^Count from drone imagery; ^#^Count from ground or ship-based photography.

**Figure 2:**
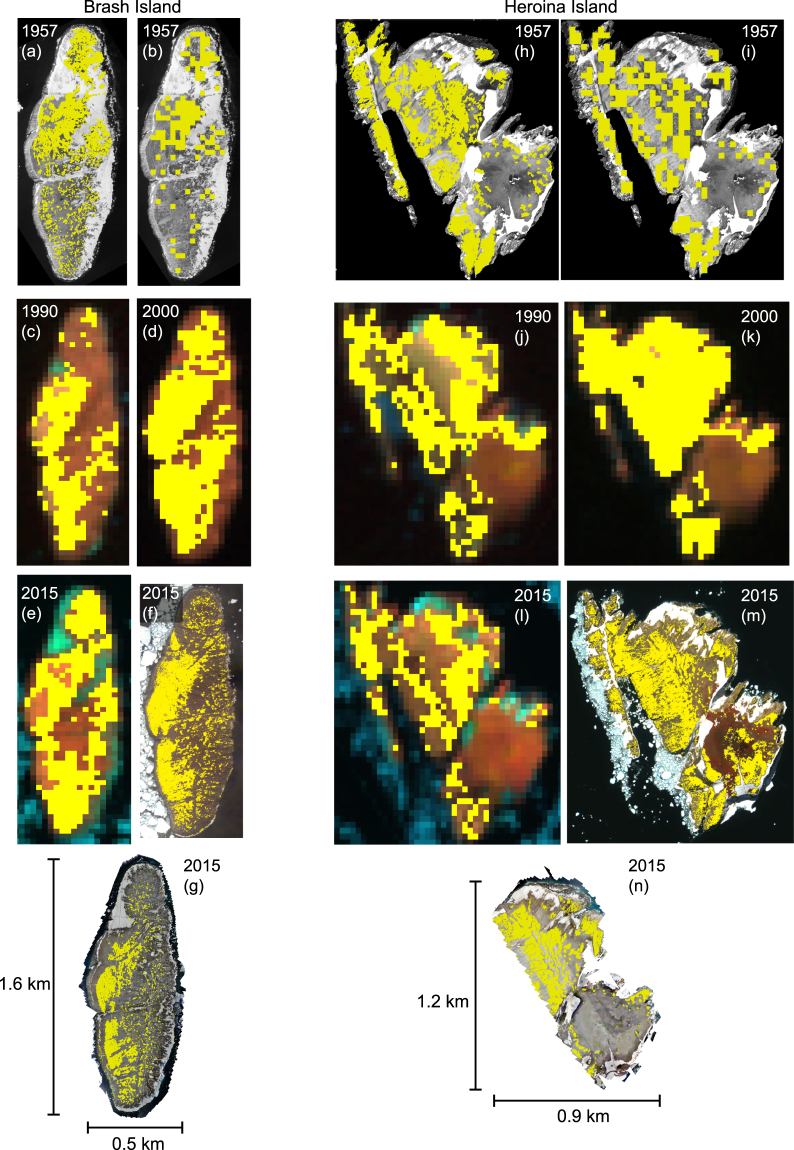
Guano areas (yellow) identified on Brash Island (at left) and Heroína Island (at right) from 1957 to present day. (**a**) and (**h)** manually classified from aerial imagery from 1957; (**b)** and (**i)** manually classified from aerial imagery from 1957 and reduced to 30 m cells for comparison with Landsat; (**c**) and (**j**): Landsat-4 in 1990 classified as described in Methods; (**d)** and (**k)** Landsat-7 in 2000 classified as described in the Methods; (**e)** and (**l)** Landsat-8 in 2015 classified as described in the Methods; (**f)** Worldview-2 image taken 19 February 2016 classified as described in the Methods (©2018, DigitalGlobe); (**m)** Worldview-2 image taken 2 December 2015 classified as described in the Methods (©2018, DigitalGlobe); (**g)** and (**n)** nests that were retained by the spatial filter marked as yellow dots overlaid on UAV imagery from ground survey described in this manuscript. Panels a, b, f, g, h, i, m, and n displayed using ESRI ArcMap 10.0 (http://desktop.arcgis.com/en/arcmap/); Panels c, d, e, h, k, and l displayed using ENVI 5.4 (https://www.harris.com/solution/envi).

Visual comparison of available aerial, satellite, and unmanned aerial vehicle (UAV) images suggests that the area occupied by Adélie penguin colonies on the Danger Islands has remained stable or has modestly increased over the last 60 years, though our inference regarding dynamics is unavoidably limited by the lack of imagery between 1957 and 1990 (Fig. 2). A previous ground-based estimate^28^ of 285,000–305,000 Adélie penguin nests on Heroína Island in 1996/97 is remarkably consistent with our updated estimate of 292,363 nests. The consensus of all the data considered in this analysis strongly suggests that the Danger Islands have remained roughly stable since the earliest records of the 1950s, in stark contrast to declines seen along the WAP.

In addition to Adélie penguins, we found several populations (>100 nests) of gentoo penguins (*P*. *papua*), particularly at Brash Island, and one small population (27 nests) of chinstrap penguins (*P*. *antarctica*) at Heroína Island. Additional information on flying birds and marine mammal observations collected during this survey are included in Supplementary Information Table S1.

## Discussion

This survey provides the first estimates of penguin abundance for this portion of the AP region and the first direct ground survey of the Danger Islands beyond Heroína Island, which was last surveyed in 1996/97. Our estimate is more than three times the abundance estimated by an earlier survey^8^, largely because several colonies, not known to exist at the time, were missed entirely. We find the Adélie penguin colonies on Heroína Island and Beagle Island are the third and fourth largest Adélie penguin colonies in the world^21^, respectively, and represent the easternmost Antarctic colonies (54°W) of all three pygoscelid penguins until 45°E. Our ground- and UAV-derived survey provides important validation of satellite imagery as a tool for the discovery of new penguin colonies, and demonstrates how satellite imagery and field expeditions can be used in concert to track penguin biogeography and long-term trends. Our discovery of a major hotspot of abundance in the Danger Islands is important for our understanding of the global distribution of the Adélie penguin, and should be considered in the development of future conservation measures such as Antarctic Specially Protected Areas (ASPAs) or Marine Protected Areas (MPAs).

At a regional scale this survey increases the total estimated abundance of Adélie penguins in CCAMLR subarea 48.1 by 68%. This dramatic increase in the number of known Adélie penguin breeding pairs radically changes our estimates of krill predation in the Northern Weddell Sea, in a portion of 48.1 that, notably, has not experienced the levels of krill fishing seen elsewhere along the Antarctic Peninsula^25,32^. The Adélie penguins found in the Danger Islands are among those penguins breeding north of the “Adélie gap,” a stretch of the coast along the WAP roughly 400 km long from the Adélie colonies on the southwestern shore of Anvers Island to the south, to Nelson Island in the South Shetland Islands and nearly the tip of the AP to the north^33,34^. This gap is devoid of breeding Adélie penguins and geographically differentiates those Adélie penguins to the south from those in the north in both summer and winter foraging habitat^34^. The new abundance estimate for the northern portion of subarea 48.1 highlights the spatial structure of predator abundance, and reveals an area of high abundance distinct from the better surveyed coastline of the WAP. Accordingly, an updated understanding of predator distributions, particularly the location of major abundance hotspots, may have implications for the management of prey resources^35,36^.

While our inference on past trends is unavoidably limited by the lack of prior ground surveys, our analysis of the available imagery suggests that Adélie penguin colonies in the Danger Islands have not suffered the net declines seen on the WAP, where some colonies have declined by an order of magnitude or even disappeared completely^14,37,38^. Our findings are consistent with recent modelling work^39^ showing that the warming of the WAP has followed a west-to-east pattern, such that the Danger Islands have been largely spared the environmental changes experienced by the South Shetland Islands and the northern portion of the WAP. In particular, the Weddell Sea has not experienced the significant loss of sea ice seen in the Bellingshausen Sea, and instead shows slight gains over the past several decades^40,41^, providing more consistent foraging habitat for pagophilic species such as the Adélie penguin. We recognize, however, that while the evidence for stability from 1990-present is well supported by the available imagery, the evidence cannot rule out sequential and roughly compensatory periods of increase and decrease in the earlier period (1957–1990)^42^.

Given the large number of Adélie penguins breeding in the Danger Islands, and the likelihood that the northern Weddell Sea will remain suitable for Adélie penguins longer than the rest of the Antarctic Peninsula region, we suggest the Danger Islands should be strongly considered for further protection, either through an extension of the proposed Weddell Sea MPA that falls just to its south or by way of an MPA in the Western Antarctic Peninsula^43,44^. Like the Ross Sea, the northern Weddell Sea represents an Adélie penguin hotspot of significant conservation value as a potential refugium under climate change. By establishing the distribution and abundance of penguins in this region, we hope to highlight its importance to regional and global populations, and encourage more regular monitoring of the region.

## Methods

We define the Danger Islands as including (from north to south): Brash Island, Heroína Island, Comb Island (also known as Peine Island), Beagle Island, Platter Island (Plato Island), Darwin Island, and Earle Island (Fig. 1). Dixey Rock and Scud Rock are also located in this area and were also surveyed. These islands range from generally low and flat (Platter Island), to sheer cliff faces (Darwin and Comb Islands), with most containing a mix of steep scree slopes, flat areas, and cliffs. The islands are composed of intrusive igneous rocks, predominantly feldspar-rich gabbro, that were formed during the late Cretaceous and are of similar age to the plutonic rock formations at the tip of the Antarctic Peninsula^45^. Data from this region are sparse, but during the last glacial maximum these islands may have been glaciated until around 6000 years before present (bp)^46^. While the Holocene occupation history of penguins on the Danger Islands is currently undescribed, radiocarbon-dated remains from other northern Antarctic Peninsula breeding sites indicates a relatively recent (~600 bp) advent of breeding populations^47^.

### Field survey

Surveys were conducted from the M/V *Hans Hansson* from December 9–18, 2015. We used a variety of survey methods on each island depending on conditions and time ashore, including one or more of the following methods: (1) manually counting individual nests, (2) counting individual nests in panoramic photos taken from the ground or the vessel, and (3) counting individual penguins from photographs captured by UAV. The combination of these methods allowed for efficient data collection with opportunities for cross-validation of survey methods. The precision of census counts varied by island (Table 1), and island-specific error estimates were propagated to the archipelago-wide confidence intervals for total abundance.

The timing of our expedition was ideal in terms of penguin phenology^48^, and the surveyed colonies were dominated by individual penguins incubating well-established nests. Our estimates of abundance, therefore, represent a count of all ‘actively’ incubated nests on each island. Active nests were those occupied by a penguin, noting that the presence of eggs or chicks in the nest cannot be determined from aerial photography. To facilitate counting, islands were divided based on the natural boundaries between “sub-colonies,” or naturally-occurring, discrete groups of penguin nests. For those sub-colonies too large to count accurately in their entirety, divisions were made based on natural markers within the sub-colony or, in their absence, using brightly coloured rope laid between nests; such subsections were counted individually. To ensure accuracy, each division was counted three times and these three counts were required to agree within 5% of their mean. If counts did not agree, divisions were further subdivided until three subsequent counts did agree within 5% of the mean. This 5% accuracy threshold corresponds to the ‘N1’ level of precision described by Croxall and Kirkwood^49^ and used regularly to report penguin abundance in the Antarctic (e.g., refs^28,33,50^).

Full, site-wide counts for Adélie penguins were conducted at Platter and Earle Islands, the latter of which was also surveyed by UAV (Table 1). Manual counts of well-defined Adélie penguin sub-colonies were conducted at Heroína, Brash, and Beagle Islands as a validation of counts based on UAV photographs. Where present, we conducted site-wide counts of gentoo penguins and chinstrap penguins as well (Table 1). Adélie penguin populations at Comb and Darwin Islands were counted from images taken on the ground or from vessels offshore of the islands using Adobe Photoshop’s count tool. On all islands, the presence of other bird or mammal species was noted opportunistically (Supplementary Information Table S1).

All research was conducted with under the approval of Stony Brook University’s Institutional Animal Care and Use Committee (237420), Woods Hole Oceanographic Institution’s Institutional Animal Care and Use Committee (18958), and following ethical review by the University of Oxford. This expedition was permitted under Antarctic Conservation Act Permit ACA 2016-011. An Initial Environmental Evaluation was approved by the US Environmental Protection Agency on 1 December 2015, and Advance Notification provided to the US Department of State.

### UAV-based survey

UAV surveys were performed with a DJI Phantom 3 quadcopter using its stock 1.2 Megapixel camera. The UAV was flown either manually or automatically using the mission planning software Map Pilot App to generate image coverage of each island with at least 70% overlap between images. Following the suggestions laid out by ref.^51^, a minimum height above ground of 25 m was set for all flights to avoid disturbance to wildlife, and a maximum height above ground of 45 m was selected to maintain image quality for penguin identification. The geotagged imagery collected with the UAV was post-processed using the commercial photogrammetry software Photoscan (Agisoft LLC, St. Petersburg, Russia) which generated full, georeferenced orthomosaics, a top-down view of the island – in which each pixel corresponds to a fixed physical dimension – of the surveyed islands and their penguin colonies.

**Figure 3:**
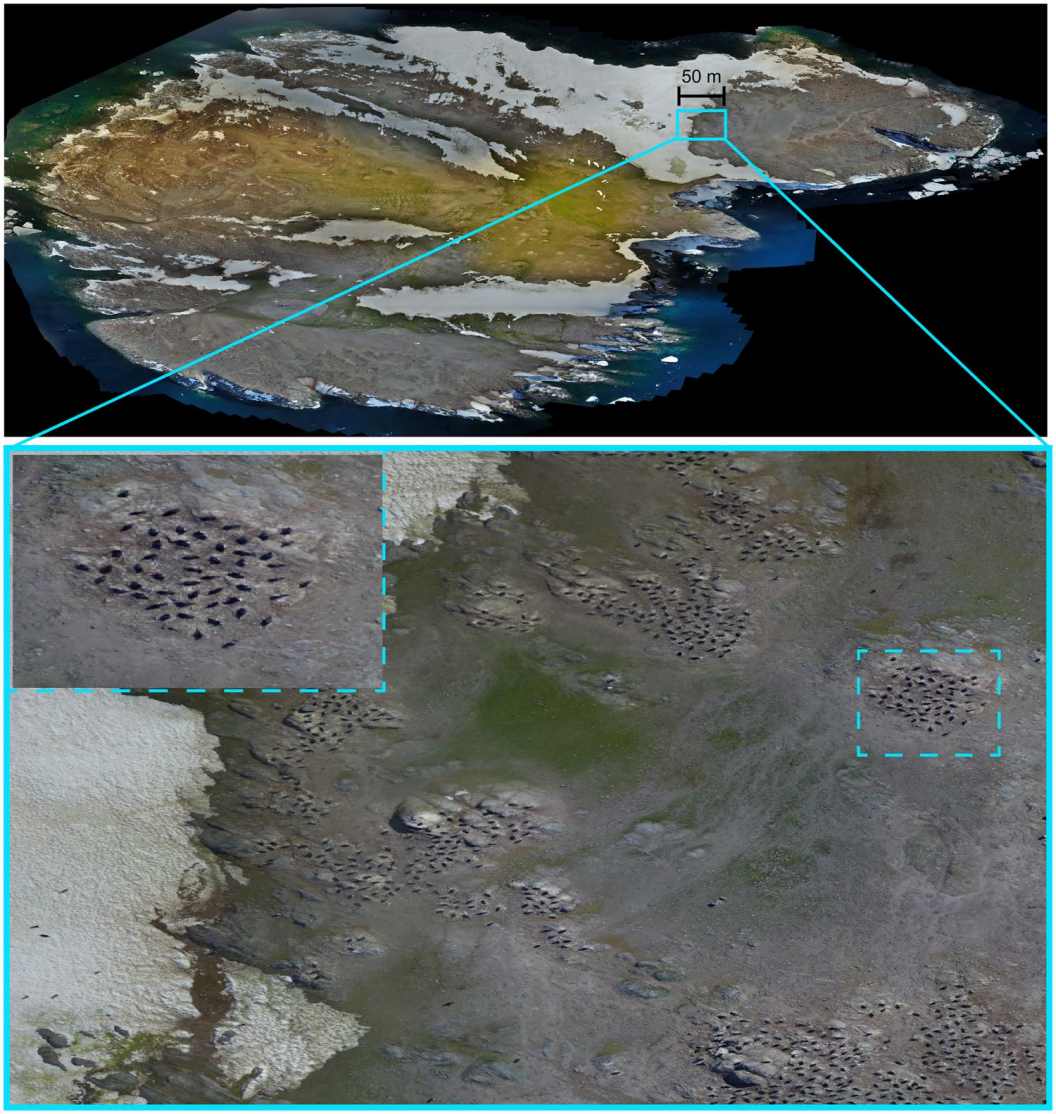
UAV orthomosaic image of Brash Island (above), with examples of zoomed-in penguin rookeries (below), displayed using ESRI ArcMap 10.0 (http://desktop.arcgis.com/en/arcmap/).

Brash, Earle, Beagle, and Heroína Islands were surveyed using composite panoramic images captured by the UAV (example in Fig. 3). The timing of our survey was ideal for capturing incubating penguins on the nest and the imagery was, in the overwhelming majority of cases, unambiguous with respect to penguins that were incubating versus walking through the colony or from the ocean. To automatically identify and count the number of occupied nests in the UAV orthomosaics we used a Deep Neural Network (DetectNet) implemented in the open source software NVIDIA DIGITS (NVIDIA Corporation, Santa Clara, CA). DetectNet is based on the GoogLeNet image classification framework^52^ and is specifically designed to locate multiple objects of the same type within an image, making it well-suited to the task of detecting penguins in aerial imagery. The DetectNet network was trained to detect penguins using 512 × 512 sub-images selected from the orthophotos of the four islands being analysed and manually annotated with penguin locations. The images were split into two groups, one for training the network and one for validation, with 160 images and 1237 penguins in the training group and 93 images and 673 penguins in the validation group. The manually-labelled training data constituted 0.18% of the imaged area and 0.34% of the imaged penguins providing a massive decrease in manual labour required. Once trained, full island detection was performed by splitting the orthophotos into 512 × 512 sub-images which were run through the trained detector in DIGITS.

False positives generated by this automated nest detection algorithm were comprised of both individual non-nesting penguins and artefacts, such as rocks and shadows, that appear visually similar to nesting penguins. While nesting penguins are highly spatially structured, with strong attraction between individuals^53^, the false positives are largely spatially unstructured. We therefore applied an additional spatial filter to the detections, retaining nests based on the distribution of nearest neighbours. Points passing through this spatial filter are classified as unambiguous penguin nests and are retained; points rejected at this stage are comprised of false positives and a much smaller number of isolated nests incorrectly rejected by the filtering process. We validated our nest detections by manually counting a selection of each of the four islands surveyed by UAV (Brash, Earle, Beagle, Heroína), and created a simple linear regression model to estimate the number of nests based on the number detected (Supplementary Methods S1). These site-specific linear models allowed us to correct for any site-specific differences in the performance of the detection algorithm. Based on this analysis, we have classified our automated counts as $\pm 10 \%$ accuracy (i.e. an ‘N2’ count), though we note that the average difference between the automated nest counting of UAV imagery and an *in situ* ground count of the same portion of the colony was only 0.6% and so our estimates may be even more precise than suggested by an ‘N2’ designation.

### Historical aerial imagery

To understand the potential population trajectory of penguins in the Danger Islands region, the spatial extent of current penguin colonies can be qualitatively compared to historical aerial photographs (Fig. 2). We selected cloud-free photographs from the Falkland Islands Dependencies Aerial Survey Expedition (FIDASE) for Heroína^54^ and Brash^55^ islands. These islands were chosen as the FIDASE archive contained quality photographs shot nearly on nadir and both had been fully photographed by the UAV. The islands were photographed in black and white on Jan. 31, 1957 at an altitude of 4115 m to a scale of 1:27000, and digitally scanned by the U.S. Geological Survey. Both island frames were georeferenced to WorldView-2 satellite images and divided into polygons using segmentation algorithms tuned to provide a segment size reasonable for further analysis using Quantum GIS^56^ (QGIS). In both cases, segments were manually classified by a skilled observer as “guano” to designate recent guano deposition, “guano-like” to designate areas thought to be guano but with less certainly, “old guano” to designate areas of guano accumulation not necessarily associated with active nesting, and “non-guano,” a classification encompassing rock, water, snow, and all other substrates not covered by guano (Fig. 2).

### Landsat satellite imagery analysis

Adélie penguin colonies identified in Fig. 2 were retrieved from Landsat imagery based on the algorithm described by refs^31,57^. The retrieval algorithm was originally developed for a single sensor and was modified in this case to operate on cross-calibrated data from Landsat-4 (imagery date 1990 in Fig. 2), −7 (2000) and −8 (2015). Cross-calibration among sensors was performed by calculating the mean difference of similar bands from Landsat-4 and-8 imagery compared to Landsat-7, and then adjusting the band values based on the mean differences in each spectral band. The algorithm was then applied to the cross-calibrated imagery to classify Adélie penguin colony areas.

### High-resolution commercial imagery

Areas of guano staining were manually identified in high resolution satellite imagery (Fig. 2 panels f and m). A selection of unambiguous pixels within the guano stains were used to select other pixels (using Adobe Photoshop) similar in colour, and areas were added and removed manually based on manual interpretation of the imagery combined with auxiliary information from the UAV imagery mosaics.

### Data availability

All Landsat data are archived by the U.S. Geological Survey. The data can be acquired at no cost via EarthExplorer (https://earthexplorer.usgs.gov/) and several other on-line tools hosted by the USGS. High resolution commercial satellite imagery (e.g., Worldview, Quickbird) is subject to a licensing agreement with Digital Globe, Inc. and inquiries should be directed to the Polar Geospatial Center (www.pgc.umn.edu/). UAV image mosaics, ArcGIS shapefile layers for penguin nests, and additional photographic data collected during this expedition may be obtained from the authors on request.

## Electronic supplementary material

Supplementary Info
