# Supplementary material for: Multi-modal survey of Adélie penguin mega-colonies reveals the Danger Islands as a seabird hotspot
Source: Sci Rep. 2018 Mar 2;8:3926. doi: 10.1038/s41598-018-22313-w (PMC5834637; doi:10.1038/s41598-018-22313-w)
Supplement: Supplementary file 1 — Supplementary Info [file 41598_2018_22313_MOESM1_ESM.pdf]

Supplementary Information for: Multi-modal survey of Adélie penguin mega-colonies reveals the Danger Islands as a seabird hotspot

Authors

Alex Borowicz[1], Philip McDowall[1], Casey Youngflesh[1], Thomas Sayre-McCord[2,3], Gemma Clucas[4, 5], Rachael Herman[1,7], Steven Forrest[6], Melissa Rider[6], Mathew Schwaller[1], Tom Hart[4], Stéphanie Jenouvrier[8, 9], Michael J. Polito[7], Hanumant Singh[2], Heather J. Lynch[1]

Author affiliations

[1] Department of Ecology and Evolution, 113 Life Sciences, Stony Brook University, Stony Brook, NY 11794, United States. [2] Department of Applied Ocean Physics and Engineering, Woods Hole Oceanographic Institution, Woods Hole, MA 02543, United States. [3] Department of Mechanical Engineering, Massachusetts Institute of Technology, Cambridge, MA 02139, United States. [4] Department of Zoology, South Parks Road, Oxford, OX1 3PS, United Kingdom. [5] Natural Resources and the Environment, James Hall, University of New Hampshire, Durham, NH 03824, United States. [6] Antarctic Resource, Inc., 303 S. Broadway, Suite 200-190, Denver, CO 80209, United States. [7] Department of Oceanography and Coastal Sciences, Louisiana State University, Baton Rouge, 70803, United States. [8] Biology Department, Woods Hole Oceanographic Institution, Woods Hole, MA, United States. [9] Centre d'Etudes Biologiques de Chizé, UMR 7372 Centre National de la Recherche Scientifique/Univ La Rochelle, Villiers en Bois, France. Correspondence and requests for materials should be addressed to H.J.L (email: heather.lynch@stonybrook.edu)

Supplementary Methods S1

This supplement provides R code to estimate nests for each site. It demonstrates site-specific differences in the errors of automated counting given manual counts, and provides counts for each island based on the island-specific correction for bias in the detection algorithm. Island-specific biases in the detection algorithm arise from differences in topography and geology of the different islands, as well as sun angle during the UAV image collection. In the code that follows, we read in manual counts of quadrats within each site, and the results of the automated nest-counting algorithm within those same quadrats. We use a simple no-intercept linear model to estimate true nests given the number counted in using the automated counting algorithm.

In the first step we simply define a function that counts up all the points (from either manual counting or from automated counting) within the validation quadrats.

```
# Data frame in which to put corrected counts and prediction intervals
results=data.frame(site=c('hero','brash','earl','beagle'),initial_count=0,lower=0,corrected=0,upper=0)

#Function to count points from manual count data and automated count data,
#And also takes in the grid quadrats used to delineate counts.
quad_count <- function(manual_points,auto_points,quadrats){
  counts = data.frame('quad' = quadrats,'manual'=0,'auto'=0)
  row = 1
  for(quad in quadrats){
    manual_count = sum(manual_points$FID_2 == quad,na.rm = TRUE)
    auto_count = sum(auto_points$FID_2 == quad,na.rm = TRUE)
    counts[row,] = c(quad,manual_count,auto_count)
    row=row+1
  }
  return(counts)
}
```

For each island, we simply load in the data on the locations of nests identified manually as well as those identified by the automated process described in the manuscript. We then fit a linear (no intercept) regression and plot that regression line on the original counts, along with the upper and lower prediction intervals and the 1:1 line (for reference).

Heroina

```
#Heroina Quadrats
hero_samples=scan("C:/Work/Penguins/Danger Islands expedition/Dangers_Analysis/Heroina/tables/quad_samples.txt")
#Read in data - Manually-counted points
manual_points <- read.csv("C:/Work/Penguins/Danger Islands expedition/Dangers_Analysis/Heroina/tables/manual_points_final.csv")
#Automated counter points
auto_points <- read.csv("C:/Work/Penguins/Danger Islands expedition/Dangers_Analysis/Heroina/tables/auto_points_final.csv")
# Points-to-numbers function
counts = quad_count(manual_points,auto_points,hero_samples)
#Write the points-to-counts to a csv file
write.csv(counts,"C:/Work/Penguins/Danger Islands expedition/Dangers_Analysis/Heroina/tables/hero_counts.csv")

#Linear model of manual counts to automated counts
#Taking in output of the function above
hero_model = lm(manual ~ auto + 0,data=counts)

# Set auto count data as the test data
test_data=data.frame('auto'=1:max(counts$auto))
# Create prediction intervals
preds = predict(hero_model,newdata = test_data,interval = c("prediction"),level = 0.95,  type="terms")
# Plotting
plot(x=test_data$auto,y=preds$fit,type='l',ylab='Manual Count',xlab='Automated Count',main='Heroina Count Model')
points(x=counts$auto,y=counts$manual,col=rgb(0,0,0,0.5),pch=16) #Plot points
lines(test_data$auto,preds$lwr,col="red",lty=2) #Pred. intervals added
lines(test_data$auto,preds$upr,col="red",lty=2)
abline(0,1,col='green') #Add a 1:1 line for reference
```

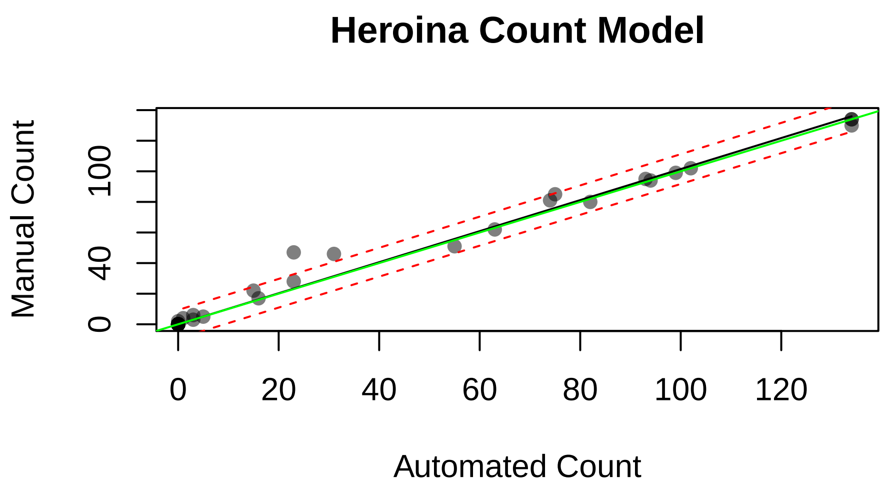


```
#Final corrected counts
hero_final = predict(hero_model,newdata = data.frame('auto'=nrow(auto_points)),interval = c("prediction"),level = 0.95,  type="response")
#Store the results
results[1,]= c('site'='hero','count'=nrow(auto_points),'lower'=hero_final[2],'corrected'=hero_final[1],'upper'=hero_final[3])
```

The total automated count is 5.4% smaller than the total manual count across these validation quadrats.

Brash Island

```
# Read in data
brash_samples = scan("C:/Work/Penguins/Danger Islands expedition/Dangers_Analysis/Brash/tables/quad_samples.txt") #quadrats
#Manually-counted points
manual_points <- read.csv("C:/Work/Penguins/Danger Islands expedition/Dangers_Analysis/Brash/tables/manual_points_final.csv")
#Points from automated counter
auto_points <- read.csv("C:/Work/Penguins/Danger Islands expedition/Dangers_Analysis/Brash/tables/auto_points_final.csv")
# Count the points
counts = quad_count(manual_points,auto_points,brash_samples)
# Write points-to-counts to csv
write.csv(counts,"C:/Work/Penguins/Danger Islands expedition/Dangers_Analysis/Brash/tables/brash_counts.csv")
#Linear model
brash_model = lm(manual ~ auto + 0,data=counts)
# Auto count as test data
test_data=data.frame('auto'=1:max(counts$auto))
#Prediction interval
preds = predict(brash_model,newdata = test_data,interval = c("prediction"),level = 0.95,  type="terms")
# Plotting
plot(x=test_data$auto,y=preds$fit,type='l',ylab='Manual Count',xlab='Automated Count',main='Brash Count Model')
points(x=counts$auto,y=counts$manual,col=rgb(0,0,0,0.5),pch=16) #Add points
lines(test_data$auto,preds$lwr,col="red",lty=2) #pred. intervals added
lines(test_data$auto,preds$upr,col="red",lty=2)
abline(0,1,col='green')  #Add 1:1 line for reference
```

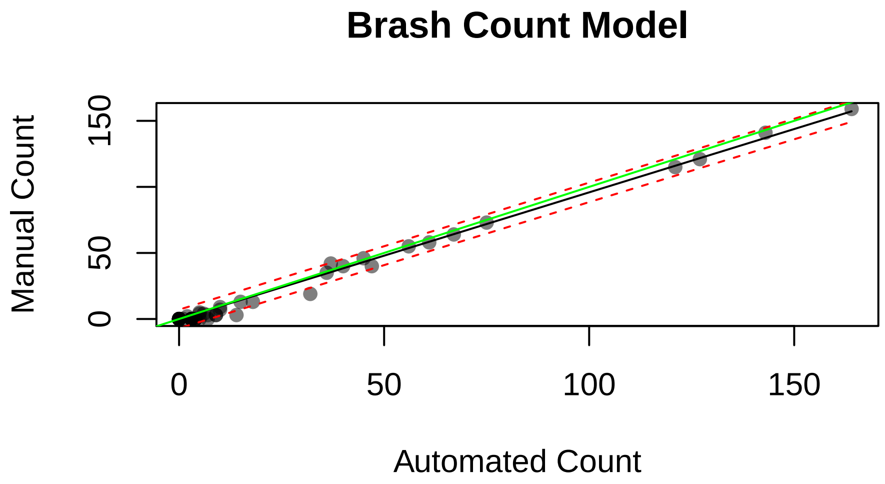


```
# Final corrected counts
brash_final = predict(brash_model,newdata = data.frame('auto'=nrow(auto_points)),interval = c("prediction"),level = 0.95,  type="response"
# Store results
results[2,] = c('brash',nrow(auto_points),brash_final[2],brash_final[1],brash_final[3])
```

The total automated count is 10.5% larger than the total manual count across these validation quadrats.

Earl

```
# Read in quadrats
earl_samples = scan("C:/Work/Penguins/Danger Islands expedition/Dangers_Analysis/Earl/tables/quad_samples.txt")
# Read in data
# Manually-counted points
manual_points <- read.csv("C:/Work/Penguins/Danger Islands expedition/Dangers_Analysis/Earl/tables/manual_points_final.csv")
# Points from automated counter
auto_points <- read.csv("C:/Work/Penguins/Danger Islands expedition/Dangers_Analysis/Earl/tables/auto_points_final.csv")
#Convert points to counts
counts = quad_count(manual_points,auto_points,earl_samples)
# Write counts to csv
write.csv(counts,"C:/Work/Penguins/Danger Islands expedition/Dangers_Analysis/Earl/tables/earl_counts.csv")

#Linear model
earl_model = lm(manual ~ auto + 0,data=counts)
#Auto counts as test data
test_data=data.frame('auto'=1:max(counts$auto))
# Prediction intervals
preds = predict(earl_model,newdata = test_data,interval = c("prediction"),level = 0.95,  type="terms")
#Plotting
plot(x=test_data$auto,y=preds$fit,type='l',ylab='Manual Count',xlab='Automated Count',main='Earl Count Model')
points(x=counts$auto,y=counts$manual,col=rgb(0,0,0,0.5),pch=16) #Add points
lines(test_data$auto,preds$lwr,col="red",lty=2) #Prediction intervals added
lines(test_data$auto,preds$upr,col="red",lty=2)
abline(0,1,col='green') #Add 1:1 line for reference
```

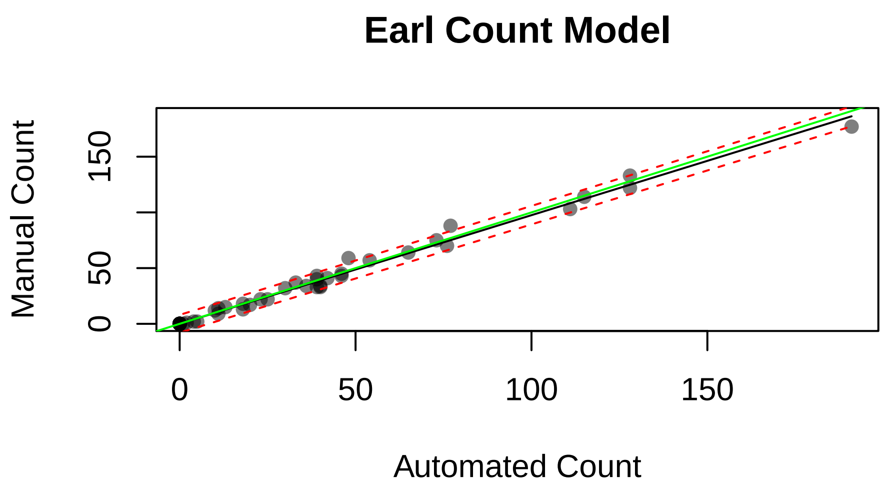


```
#Corrected counts
earl_final = predict(earl_model,newdata = data.frame('auto'=nrow(auto_points)),interval = c("prediction"),level = 0.95,  type="response")
#Store results
results[3,] =  c('earl',nrow(auto_points),earl_final[2],earl_final[1],earl_final[3])
```

The total automated count is 2.0% larger than the total manual count across these validation quadrats.

Beagle

```
#Beagle quadrats
beagle_samples<- scan("C:/Work/Penguins/Danger Islands expedition/Dangers_Analysis/Beagle/tables/quad_samples.txt")
#Read in data
#Manually-counted points
manual_points <- read.csv("C:/Work/Penguins/Danger Islands expedition/Dangers_Analysis/Beagle/tables/manual_points_final.csv")
# Points from automated coutner
auto_points <- read.csv("C:/Work/Penguins/Danger Islands expedition/Dangers_Analysis/Beagle/tables/auto_points_final.csv")
#Convert points to counts
counts = quad_count(manual_points,auto_points,beagle_samples)
#Write counts to csv
write.csv(counts,"C:/Work/Penguins/Danger Islands expedition/Dangers_Analysis/Beagle/tables/beagle_counts.csv")
#Linear model
beagle_model = lm(manual ~ auto + 0,data=counts)
#Auto counts as test data
test_data=data.frame('auto'=1:max(counts$auto))
#Predction intervals
preds = predict(beagle_model,newdata = test_data,interval = c("prediction"),level = 0.95,  type="terms")
#Plotting
plot(x=test_data$auto,y=preds$fit,type='l',ylab='Manual Count',xlab='Automated Count',main='Beagle Count Model')
points(x=counts$auto,y=counts$manual,col=rgb(0,0,0,0.5),pch=16) #Plot points
lines(test_data$auto,preds$lwr,col="red",lty=2) #Pred. intervals added
lines(test_data$auto,preds$upr,col="red",lty=2)
abline(0,1,col='green') #1:1 line for reference
```

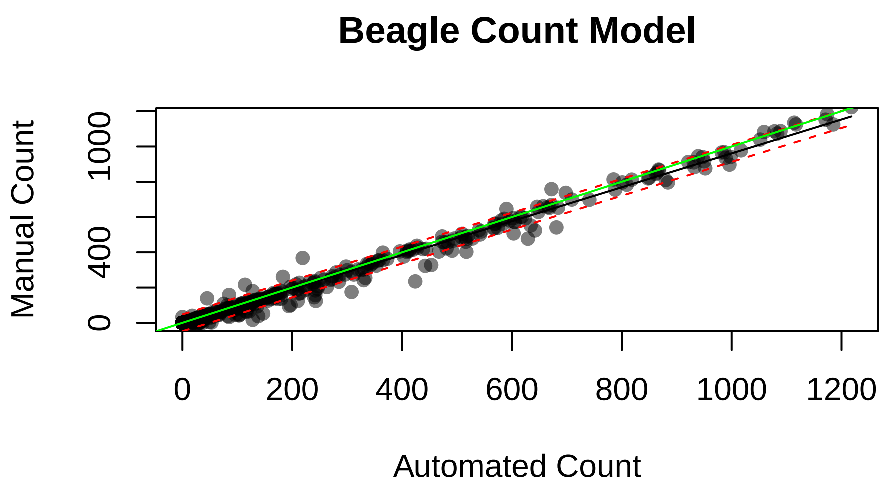


```
# Corrected counts
beagle_final = predict(beagle_model,newdata = data.frame('auto'=nrow(auto_points)),interval = c("prediction"),level = 0.95,  type="respons
# Store results
results[4,]= c(site='beagle',nrow(auto_points),beagle_final[2],beagle_final[1],beagle_final[3])
```

The total automated count is 6.2% larger than the total manual count across these validation quadrats.

Final Counts

```r
# Show final corrections for each island with intervals
print(results)
```

```
##     site initial_count            lower        corrected            upper
## 1   hero        288157 284543.558689769 292363.494664525 300183.430639281
## 2  brash         99041 92802.6610383077 94950.7202825562 97098.7795268047
## 3   earl         21611 20609.9509490126 21071.0198827591 21532.0888165055
## 4 beagle        296105  282733.95332708 284535.493797482 286337.034267883
```

Supplementary Table S1: List of species present in the Danger Islands. B=Verified as breeding, I=Individuals present, NB=Verified as not breeding, – =Not observed or No data.

**Species**	**Beagle**	**Brash**	**Comb**	**Darwin**	**Dixey Rock**	**Earle**	**Heroina**	**Platter**	**Scud Rock**
**Birds**									
Adélie penguin (*Pygoscelis adeliae*)	B	B	B	B	NB	B	B	B	NB
Gentoo penguin (*Pygoscelis papua*)	NB	B	B	–	NB	B	B	B	NB
Chinstrap penguin (*Pygoscelis antarctica*)	NB	NB	NB	–	NB	NB	B	NB	NB
Antarctic shag (*Phalacrocorax atriceps*)	NB	NB	–	–	NB	B	I	B	NB
Skua species (*Stercorarius spp.*)	–	I	I	B	–	B	B	I	–
Southern giant petrel (*Macronectes giganteus*)	NB	I/NB	NB	–	–	NB	I/NB	I/NB	–
Cape (pintado) petrel (*Daption capense*)	–	–	–	–	–	–	B	B	–
Snow petrel (*Pagodroma nivea*)	–	–	B	–	–	I	I	–	–
Wilson's storm petrel (*Oceanites oceanicus*)	–	B	–	–	–	–	I	I	–
Kelp gull (*Larus dominicanus*)	–	B	I	–	–	I	I	I	–
Snowy sheathbill (*Chionis albus*)	–	B	B	I	–	B	B	B	–
Antarctic tern (*Sterna vittata*)	–	–	I	–	–	–	I	–	–
**Seals**									
Weddell seal (*Leptonychotes weddelli*)	–	–	I	–	–	I	I	I	–
